# Cigarette smoke-induced gasdermin D activation in bronchoalveolar macrophages and bronchial epithelial cells dependently on NLRP3

**DOI:** 10.3389/fimmu.2022.918507

**Published:** 2022-08-15

**Authors:** Sarah Huot-Marchand, Mégane Nascimento, Elodie Culerier, Mélissa Bourenane, Florence Savigny, Corinne Panek, Cindy Serdjebi, Marc Le Bert, Valérie F. J. Quesniaux, Bernhard Ryffel, Petr Broz, Nicolas Riteau, Aurélie Gombault, Isabelle Couillin

**Affiliations:** ^1^ University of Orleans and CNRS, INEM-UMR7355, Orleans, France; ^2^ Biocellvia, Marseille, France; ^3^ Department of Biochemistry, University of Lausanne, Lausanne, Switzerland

**Keywords:** cigarette smoke, NLRP3 inflammasome, gasdermin D, lung, mice

## Abstract

Chronic pulmonary inflammation and chronic obstructive pulmonary disease (COPD) are major health issues largely due to air pollution and cigarette smoke (CS) exposure. The role of the innate receptor NLRP3 (nucleotide-binding domain and leucine-rich repeat containing protein 3) orchestrating inflammation through formation of an inflammasome complex in CS-induced inflammation or COPD remains controversial. Using acute and subchronic CS exposure models, we found that *Nlrp3*-deficient mice or wild-type mice treated with the NLRP3 inhibitor MCC950 presented an important reduction of inflammatory cells recruited into the bronchoalveolar space and of pulmonary inflammation with decreased chemokines and cytokines production, in particular IL-1β demonstrating the key role of NLRP3. Furthermore, mice deficient for *Caspase-1*/*Caspase-11* presented also decreased inflammation parameters, suggesting a role for the NLRP3 inflammasome. Importantly we showed that acute CS-exposure promotes NLRP3-dependent cleavage of gasdermin D in macrophages present in the bronchoalveolar space and in bronchial airway epithelial cells. Finally, *Gsdmd*-deficiency reduced acute CS-induced lung and bronchoalveolar space inflammation and IL-1β secretion. Thus, we demonstrated in our model that NLRP3 and gasdermin D are key players in CS-induced pulmonary inflammation and IL-1β release potentially through gasdermin D forming-pore and/or pyroptoctic cell death.

## Introduction

Among lung inflammatory diseases, chronic obstructive pulmonary disease (COPD) represents one of the most important public health issue. This illness is now the third leading cause of death in the world as predicted by the World Health Organization (WHO). COPD is characterized by chronic inflammation and mucus hypersecretion responsible for bronchial obstruction and that may lead to alveolar wall destruction and impaired lung functions (1, 2). Cigarette smoking (CS) is the major cause of COPD including emphysema with more than 95% of cases in industrialized countries (3). Inflammation can persist when smoking has been stopped and still be present even decades later. Frequent exacerbations of lung inflammation occur during respiratory infections which are characterized by impaired immune response to invading pathogens (4). Current treatments have limited efficiency (5) and thus research efforts to better understand the cellular and molecular mechanisms involved in airway inflammation leading to the pathophysiology of COPD have become a priority. COPD patients display a significant increase of airway recruited alveolar macrophages and neutrophils, which correlates with the disease severity (1, 2, 6).

Inflammasomes are cytoplasmic multiprotein complexes that orchestrate diverse functions during homeostasis and inflammation (7). Among the various inflammasomes, NLRP3 inflammasome demonstrated the most significant clinical relevance to date (8). The canonical NLRP3 inflammasome consists of the sensory protein NLRP3, the adaptor protein ASC (apoptosis-associated speck-like protein containing a CARD), and the effector protein caspase-1 (9, 10). NLRP3 activation requires a first signal resulting in transcriptional and post-transcriptional priming of NLRP3 and to pro-IL-1β, and pro-IL-18 expression and a second signal to promote NLRP3 inflammasome complex formation and subsequent caspase-1 activation, and pro-IL-1β and/or pro-IL-18 maturation (7).

The role of the NLRP3 inflammasome in CS-induced pulmonary inflammation and CS-resulting COPD is unclear. NLRP3 overexpression was reported in lungs of COPD patients and correlated with airway obstruction (11). *In vivo* studies reported increased NLRP3 and pro-IL-1β protein expression upon CS exposure in adult mice (12) or in offspring after maternal CS exposure (13). Mice exposure to cigarette smoke condensate (CSC) also activates NLRP3 inflammasome (14). In contrast, CS was shown to repress NLRP3 inflammasome-dependent innate immune response to asbestos in mice (15). In addition, cigarette smoke extract (CSE) or CSC induce activation of NLRP3 inflammasome in bronchial and alveolar epithelial cells and/or monocytic cells in *in vitro* experiments (16–18). Finally, CSE was shown to inhibit the NLRP3 inflammasome and to lead to caspase-1 activation *via* TLR4-TRIF-Caspase-8 axis in human macrophages (19).

Inflammasome-dependent activation of caspase-1 also leads to gasdermin D (GSDMD) protein cleavage thereby releasing its N-terminal domain, which forms pores in plasma and organellar membranes. GSDMD-N pore-forming protein was first described as the executor of pyroptosis, a programmed cell death dependent on inflammasome activation and as a part of the host innate immune defense allowing to kill bacteria-infected cells and restrict pathogen infection (20). Pyroptosis cell death leads to the release of pro-inflammatory cytokines and endogenous danger signals that amplify inflammation (21–23). On the other hand, GSDMD-mediated pyroptosis can be induced directly by caspase-8 or by the noncanonical inflammasome, *via* caspase-11-dependent cleavage (24). Besides macrophages, pyroptosis was also detected in neutrophils upon activation of non-canonical inflammasome leading to neutrophil extracellular trap (NET) formation dependent on GSDMD and caspase-11 (25), and in tissue cells in particular airway epithelial cells, exacerbating airway inflammation (26).

Importantly, GSDMD-forming pores at the plasma membrane were recently identified as the most important mechanism to explain IL-1β secretion, taking into account that this cytokine lacks a signal sequence preventing classical secretion through the endoplasmic reticulum/Golgi compartment (27). GSDMD pores were shown to be selective channels for cytokine release and ion fluxes, permitting passage of small molecules up to 25-50 kDa in particular of mature IL-1β and IL-18, independently of cell lysis, whereas larger proteins, such as the heterotetrameric caspase-1 p10/p20 (60 kDa), are released after lysis of pyroptotic macrophages (28). Interestingly, calcium influx through GSDMD pores promotes membrane repair and epithelial cell survival, highlighting that GSDMD activation may result in different fate (28, 29).

The NLRP3/caspase-1 GSDMD pathway was shown to be involved in the induction of human airway epithelial cell pyroptosis by CSE or nicotine, and associated with COPD progression (30, 31). However, whether GSDMD activation also contributes to CS-mediated pulmonary inflammation and/or COPD was poorly studied with mostly *in vitro* data and remains to be elucidated.

We here addressed the involvement of the NLRP3 inflammasome and gasdermin D in pulmonary cell influx and cytokine secretion upon acute or subchronic CS exposure.

Using an acute murine model of CS exposure which could mimic exacerbation phases of COPD or pulmonary diseases, already used to describe recent papers (32, 33), we showed that *Nlrp3-* or *Caspase-1/11*-deficiency or pharmacological treatment with the NLRP3 inhibitor MCC950 strongly reduced pulmonary inflammation and remodeling. Importantly, NLRP3 depletion reduced IL-1β levels in the bronchoalveolar space. Due to the pivotal role of IL-1β release in multiple inflammatory diseases, we hypothesized that GSDMD-forming pores contribute to CS-induced pulmonary inflammation. We showed for the first time that mouse acute CS exposure induces GSDMD activation in lung tissue and macrophages recruited into the bronchoalveolar space, through a NLRP3 dependent manner. In addition, using *Gsdmd*-deficient mice, we demonstrated that GSDMD is a key player in acute CS-induced lung inflammation.

## Results

### *Nlrp3* deficiency dampens pulmonary inflammation and remodeling in response to cigarette smoke exposure

**Figure 1:**
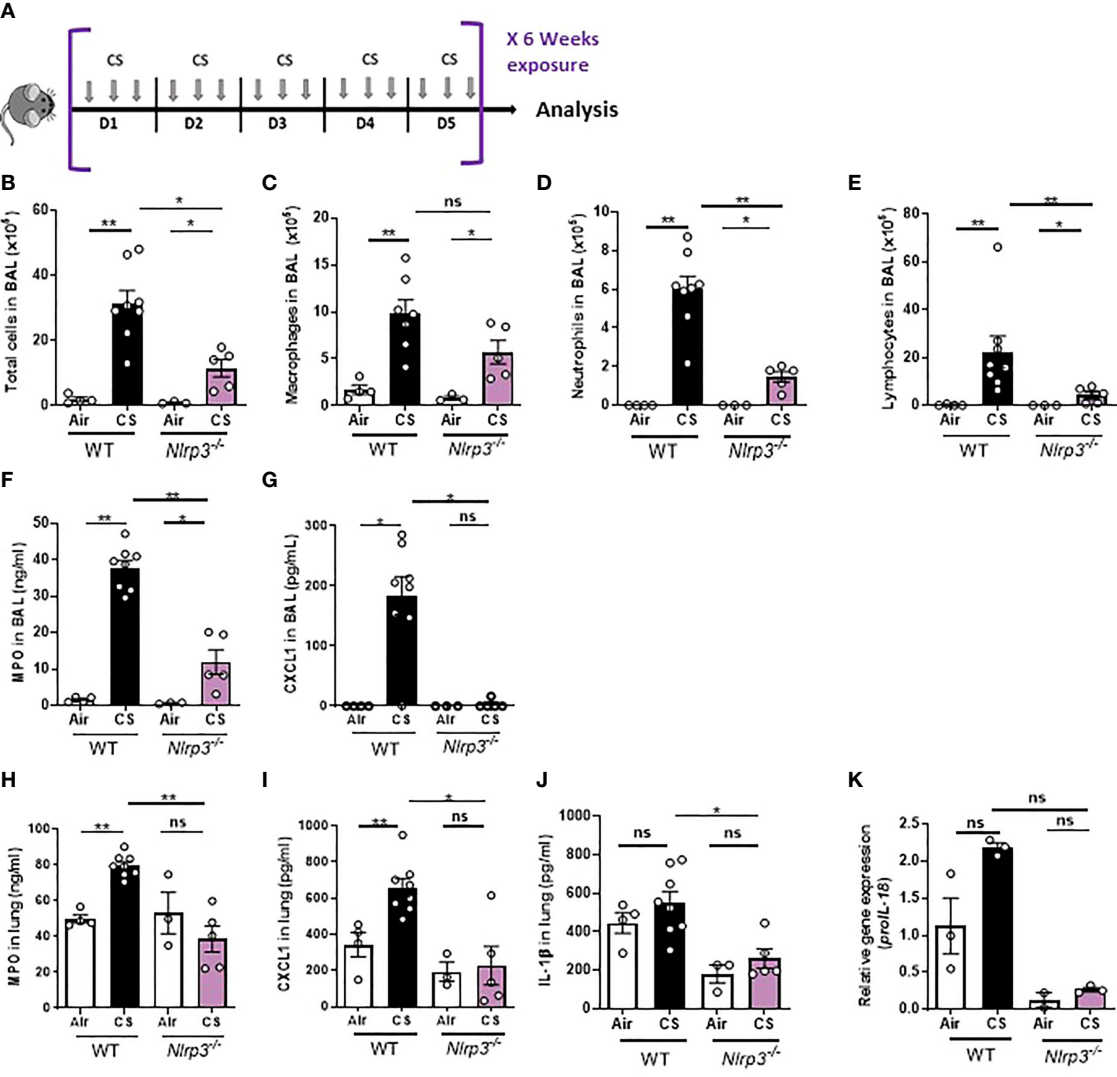
*Nlrp3* deficiency impairs inflammation to subchronic cigarette smoke (CS) exposure in mice. WT and *Nlrp3^-/-^* mice were exposed to CS or Air during 6 weeks **(A)**. CS exposure led to significant decrease counts of total cells **(B)**, macrophages **(C)**, neutrophils **(D)** and lymphocytes **(E)** into BAL of *Nlrp3^-/-^* mice compared to exposed WT mice. Levels of myeloperoxidase (MPO) **(F)**, CXCL1 **(G)** in BAL, and MPO **(H)**, CXCL1 **(I)** and IL-1β **(J)** in lung were reduced in *Nlrp3^-/-^* mice exposed to CS compared to CS WT mice. The *proIL-18* mRNA expression **(K)** was reduced in CS *Nlrp3^-/-^* mice as compared to exposed WT mice. Data are representative of one experiment and are expressed as mean values ± SEM (n= 6-9 mice per group, *p < 0.05, **p < 0.01, ns, non significant, using a Mann Whitney test).

Since the role of the NLRP3 inflammasome in cigarette smoke (CS)-induced pulmonary inflammation is not well-established *in vivo*, we analyzed the impact of *Nlrp3* deficiency using first a subchronic models of mouse CS-exposure during 6 weeks which leads to pulmonary inflammation, in comparison to chronic model of 12 weeks often used to induce experimental COPD in mice. Wild-type (WT) and *Nlrp3*-deficient (*Nlrp3* ^-/-^) mice were exposed to CS three times a day, five days a week for six weeks (**Figure 1A**). As compared to unexposed (Air) mice, subchronically CS-exposed WT mice presented increased numbers of bronchoalveolar lavage (BAL) cells (**Figure 1B**), in particular macrophages (**Figure 1C**), neutrophils (**Figure 1D**) and lymphocytes (**Figure 1E**). Importantly, neutrophils and lymphocytes recruitment into the BAL was significantly attenuated in CS-exposed *Nlrp3* ^-/-^ mice. Neutrophils play a major role in the pathogenesis developed in response to CS exposure (34, 35). As a marker of recruited neutrophils, BAL levels of myeloperoxidase (MPO) (**Figure 1F**), an enzyme present in neutrophil granules, and of the neutrophilic chemokine CXCL1 (**Figure 1G**) were elevated in CS-exposed WT mice and significantly decreased in CS-exposed *Nlrp3* ^-/-^ mice. In lung homogenates MPO (**Figure 1H**) and CXCL1 (**Figure 1I**) were also significantly reduced in CS-exposed Nlrp3^-/-^ mice compared to CS-exposed WT mice. In addition, we observed significantly reduction of IL-1β protein (**Figure 1J**) and *proIl-18* mRNA (**Figure 1K**) levels in the lungs of CS-exposed *Nlrp3* ^-/-^ mice compared to CS-exposed WT mice.

**Figure 2:**
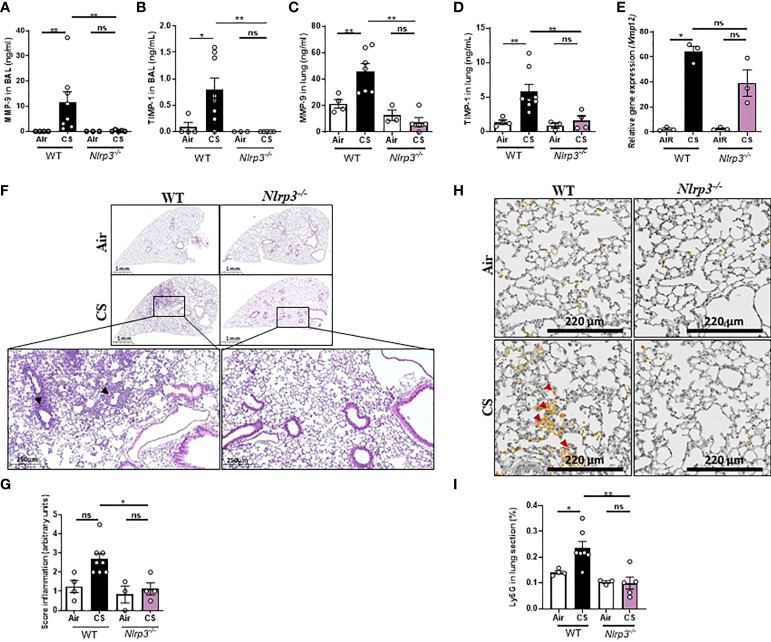
*Nlrp3* deficiency decreases remodeling factor expression and lung tissue inflammation upon subchronic cigarette smoke (CS) exposure. WT and *Nlrp3^-/-^* mice were exposed to CS or Air during 6 weeks and protein levels of remodeling factor such as MMP-9 **(A)**, TIMP-1 **(B)** in BAL, and MMP-9 **(C)**, TIMP-1 **(D)** in lung tissue were measured by ELISA whereas lung MMP-12 mRNA expression was measured by quantitative RT-PCR **(E)**. All these parameters were decreased in response to CS exposure in *Nlrp3^-/-^* mice as compared to exposed WT mice. Histological analysis of pulmonary inflammation and cell recruitment were evaluated from histology sections **(F)** and inflammation score were determined **(G)**. Ly6G immunostaining was performed on WT and *Nlrp3^-/-^* lung sections **(H)**. Percentage of Ly6G positive area per tissue section **(I)**. Data are representative of five experiments for A-G and one experiment for H and I, and are expressed as mean values ± SEM (n= 4-6 mice per group, *p < 0.05, **p < 0.01, ns, non significant, using a Mann Whitney test).

We next analyzed the balance between expression of remodeling proteases involved in the progression to COPD and their inhibitors. We focused on matrix metalloproteinase (MMP)-9, a gelatinase produced by alveolar macrophages and neutrophils that degrades extracellular matrix proteins and promotes neutrophil chemotaxis, and MMP-12 an elastase mainly produced by alveolar macrophages that contributes to alveolar damages (36) and their inhibitor tissue inhibitor of matrix metalloproteinase 1 (TIMP-1). MMP-9 and TIMP-1 protein levels (**Figures 2A, B**) in BAL and in lungs (**Figures 2C, D**) were significantly decreased in subchronically CS-exposed *Nlrp3* ^-/-^ mice as compared to WT mice. *Mmp-12* mRNA levels in lungs (**Figure 2E**) were reduced in CS-exposed *Nlrp3* ^-/-^ mice as compared to WT mice. Further, lung microsections and histology analysis showed increased cell recruitment and inflammation in lung tissue of CS-exposed WT mice compared to Air-exposed mice, which were attenuated in CS-exposed *Nlrp3* ^-/-^ mice (**Figures 2F, G**). These findings were confirmed by an increase in Ly6G labelling in lungs of CS-exposed WT mice (**Figures 2H, I**). In lungs of *Nlrp3^-/-^* mice, however, CS exposure did not affect the neutrophil count compared to Air-exposed deficient mice.

We next performed acute CS exposures during 4 days (**Figure S1A**) that confirmed reduced inflammation in *Nlrp3* ^-/-^ in comparison to WT mice. Total cells (**Figure S1B**), macrophages (**Figure S1C**) and neutrophils (**Figure S1D**) numbers in BAL were significantly attenuated in *Nlrp3* ^-/-^ mice acutely exposed to CS as compared to CS-exposed WT mice. BAL levels of MPO (**Figure S1E**), CXCL1 (**Figure S1F**), IL-1β (**Figure S1G**), as well as MMP-9 (**Figure S1H**) and TIMP-1 (**Figure S1I**) were significantly reduced in CS-exposed *Nlrp3* ^-/-^ mice compared to CS-exposed WT mice. We also observed significant reduction of CXCL1 (**Figure S1J**), IL-1β (**Figure S1K**) in lungs of CS-exposed *Nlrp3* ^-/-^ mice. Finally, MMP-9 (**Figure S1L**) and TIMP-1 (**Figure S1M**) levels in lungs of CS-exposed *Nlrp3* ^-/-^ mice were non significantly reduced as compared to WT mice. Infiltrating cells were not detected on histology slides after only 4 days of CS (data not shown). Our results demonstrate that the NLRP3 sensor is important for pulmonary inflammation and remodeling in response to CS exposure. Since our data show the role of NLRP3 in pulmonary inflammation induced by both acute and subchronic CS-exposure in mice, we next used the acute exposure model as previously (32, 33) in order to investigate the mechanism of NLRP3-mediated lung inflammation.

### Pharmacological MCC950 treatment inhibits lung inflammation induced by cigarette smoke

**Figure 3:**
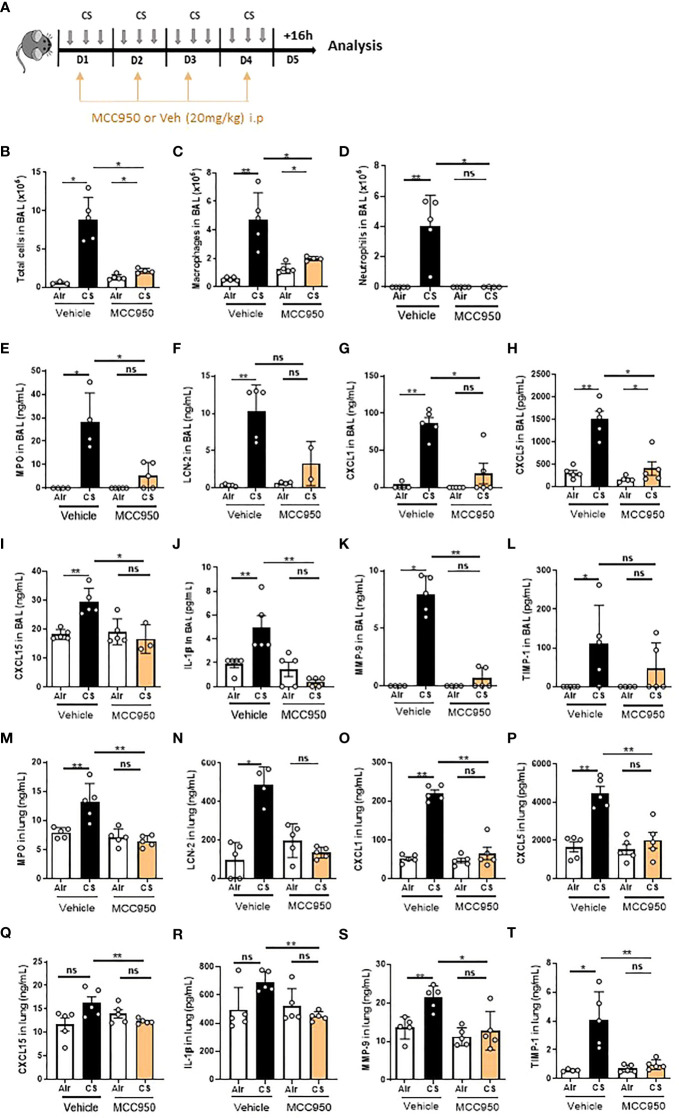
The NLRP3 inhibitor MCC950 impairs pulmonary inflammation upon acute cigarette smoke (CS) exposure. WT mice, treated or not with MCC950 (20mg/kg i.p. daily) were exposed to CS or Air during 4 days **(A)**. CS exposure led to significant decrease of total cells **(B)**, macrophages **(C)**, neutrophils **(D)** into BAL of MCC950 treated mice compared to untreated exposed WT mice. Myeloperoxidase (MPO) **(E)**, lipocaline 2 (LCN-2) **(F)**, CXCL1 **(G)**, CXCL5 **(H)** and CXCL15 **(I)** and IL-1β **(J)**, MMP-9 **(K)** and TIMP-1 **(L)** levels were measured in BAL by ELISA. All these parameters were decreased in response to CS exposure in MCC950-treated mice as compared to untreated exposed WT mice. Lung levels of MPO **(M)**, lipocaline 2 (LCN-2) **(N)**, CXCL1 **(O)**, CXCL5 **(P)**, CXCL15 **(Q)**, IL-1β **(R)**, MMP-9 **(S)** and TIMP-1 **(T)** were significantly decreased in MCC950 treated CS-exposed mice as compared to exposed WT mice. Data are representative of two experiments and are expressed as mean values ± SEM (n= 4-6 mice per group, *p < 0.05, **p < 0.01, ns, non significant, using a Mann Whitney test).

To confirm NLRP3 implication as a sensor in CS-induced pulmonary inflammation, we treated mice intraperitonealy with MCC950, a potent specific inhibitor of NLRP3 inflammasome activation (37, 38). MCC950 is active in different NLRP3-dependent inflammatory mouse models, impairing *in vivo* IL-1β production and attenuating the severity in inflammatory disease models (39). WT mice exposed to CS during 4 days were treated daily with MCC950 or vehicle (20mg/kg) (**Figure 3A**). BAL total cells (**Figure 3B**), macrophages (**Figure 3C**) and neutrophils (**Figure 3D**) were significantly reduced in MCC950-treated CS-exposed mice as compared to control group. Moreover, BAL levels of MPO levels (**Figure 3E**), of the neutrophil gelatinase-associated lipocalin (LCN)-2 (**Figure 3F**), a protein expressed by neutrophils and involved in innate immunity, of the neutrophilic chemokine CXCL1 (**Figure 3G**) and also of CXCL5 (**Figure 3H**) and CXCL15 (**Figure 3I**), two neutrophilic chemokines mainly produced by epithelial cells, were significantly reduced in BAL of MCC950-treated CS-exposed mice compared to CS-exposed WT mice. Importantly, BAL levels of the proinflammatory cytokines IL-1β (**Figure 3J**) was significantly decreased in MCC950-treated CS-exposed mice. Levels of MMP-9 (**Figure 3K**) and TIMP-1 (**Figure 3L**) were significantly and non significative reduced respectively in BAL of MCC950-treated CS-exposed mice. Furthermore, in lung tissues, levels of MPO, LCN-2, CXCL1, CXCL5, CXCL15, IL-1β, MMP-9 and TIMP-1 (**Figures 3M-T**) were significantly decreased in MCC950-treated CS-exposed compared to CS-exposed WT mice. Thus, results with NLRP3 pharmacological inhibition confirmed those obtained with NLRP3 gene deletion, showing that this receptor is involved in acute inflammation and remodeling induced by CS in mice.

### Caspase-1 and/or caspase 11 play a key role in acute CS-induced pulmonary inflammation

**Figure 4:**
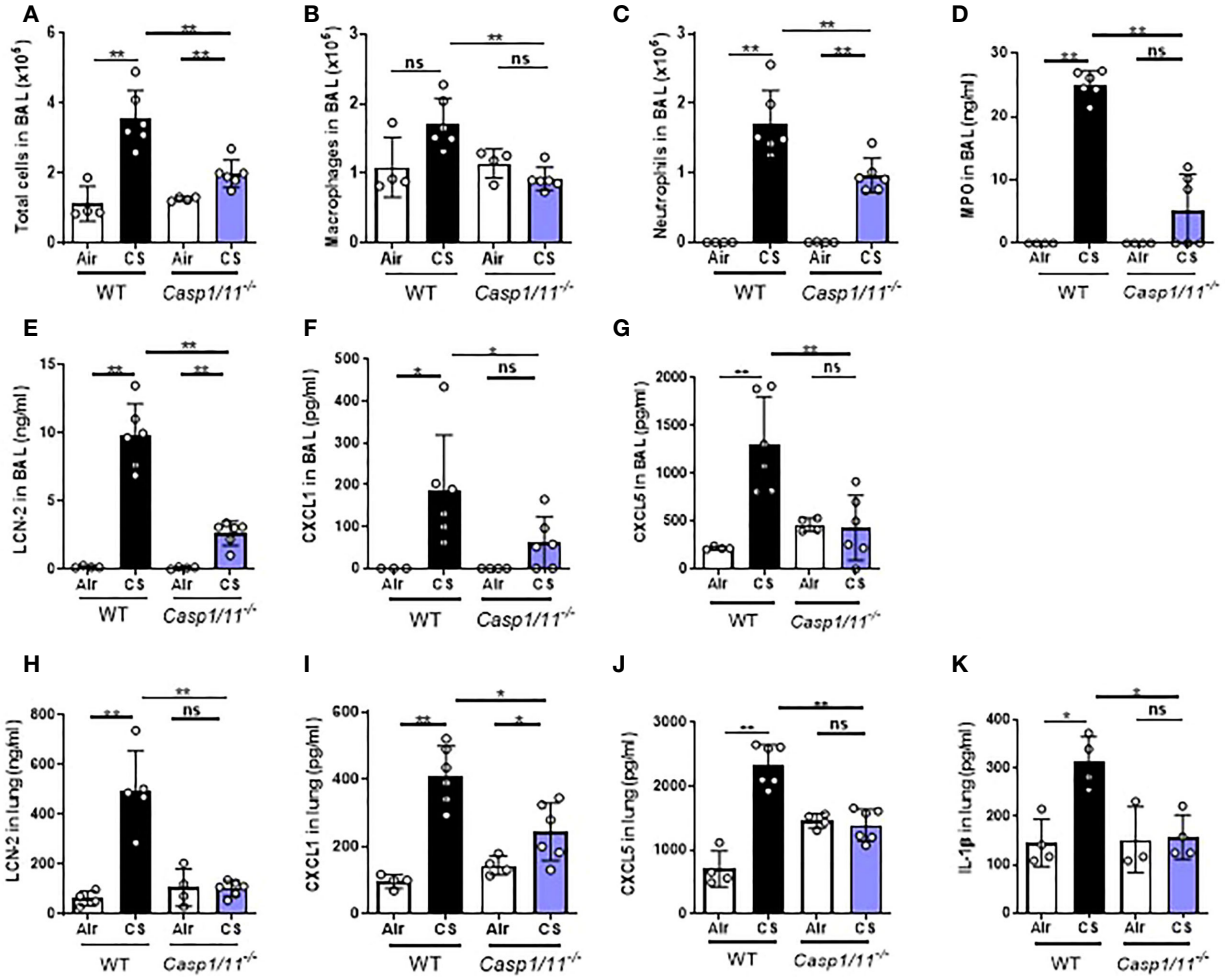
*Caspase1/11-*deficient mice (*caspase1/11^-/-^*) have reduced pulmonary inflammation after acute cigarette smoke (CS) exposure. WT and *caspase1/11^-/-^* mice were exposed to CS or Air during 4 days. Total cells **(A)**, macrophages **(B)** and neutrophils **(C)** counts and MPO levels **(D)** in BAL are shown. Lipocaline 2 (LCN-2) **(E)**, CXCL1 **(F)**, CXCL5 **(G)** levels were reduced in BAL of *caspase1/11^-/-^* CS-exposed mice. LCN-2 **(H)**, CXCL1 **(I)**, CXCL5 **(J)** and IL-1β **(K)** were also significantly reduced in lungs of *caspase1/11^-/-^* CS-exposed mice compared to CS-exposed WT mice. Data are representative of four experiments and are expressed as mean values ± SEM (n= 4-6 mice per group, *p < 0.05, **p < 0.01, ns, non significant, using a Mann Whitney test).

In order to investigate the role of the canonical and/or non canonical inflammasome in pulmonary inflammation to CS, we used mice double deficient for caspase-1 and caspase-11 *(Caspase-1/11^-/^* ^-^ *)*. Acute CS-exposure led to reduced inflammation in *Caspase-1/11^-/-^* mice in comparison to WT mice with a strong decrease in BAL total cells, macrophages and neutrophils (**Figures 4A-C**). BAL levels of MPO (**Figure 4D**) and LCN-2 (**Figure 4E**), CXCL1 (**Figure 4F**) and CXCL5 (**Figure 4G**) were significantly decreased in acutely CS-exposed *Caspase-1/11^-/-^* mice in comparison to WT. In lungs levels of LCN-2, CXCL1 and CXCL5 (**Figures 4H-J**) were also significantly reduced in *Caspase-1/11^-/-^* CS-exposed mice compared to WT CS-exposed mice. Importantly, IL-1β levels (**Figure 4K**) were significantly reduced in the lungs of *Caspase-1/11^-/-^* mice. These results indicate that either a canonical NLRP3/Caspase-1 inflammasome and/or the non-canonical inflammasome pathway (i.e. caspase-11 and NLRP3/Caspase-1 inflammasome) are involved in acute CS-induced lung inflammation.

**Figure 5:**
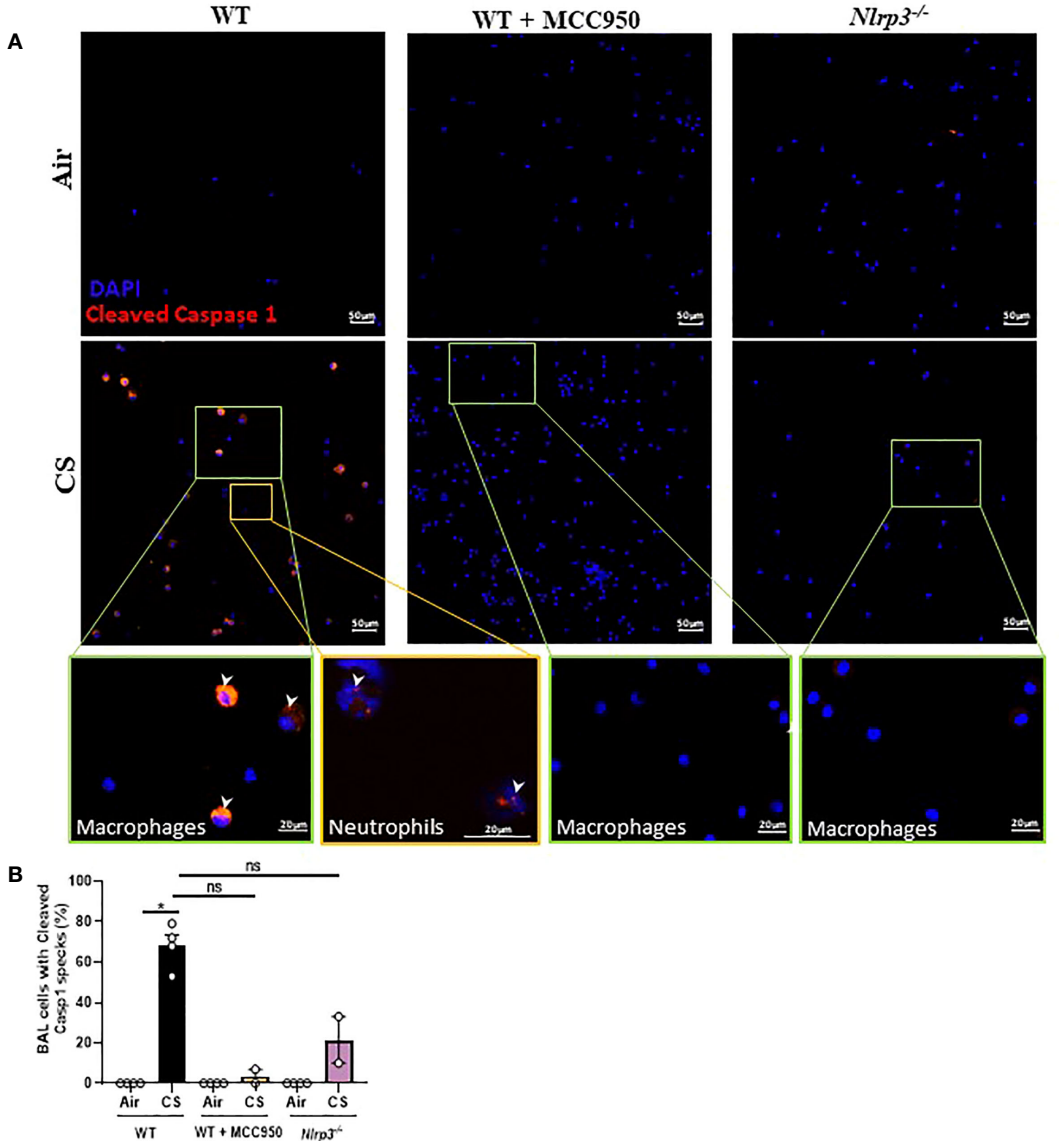
Cigarette Smoke (CS) exposure promotes caspase-1 cleavage in BAL macrophages and neutrophils in an NLRP3-dependent manner. Cleaved caspase-1 immunostaining on BAL cells collected after Air or CS exposure of WT, MCC950 WT treated mice and *Nlrp3^-/-^* mice. Cleaved caspase-1 is shown in red and nucleus in blue (DAPI) **(A)**. BAL cells fluorescence intensity quantification **(B)**. Data are representative of three experiments. Bar graph is expressed as mean ± SEM (n =2–4 mice per group, *p < 0.05 ns, non significant, using a Mann Whitney test).

In order to determine if acute CS exposure induces activation of the canonical inflammasome in alveolar macrophages and/or neutrophils recruited into the BAL, we probed for caspase-1 cleavage. Immunostaining with an antibody specific for the cleaved form (Asp 296) of murine caspase-1 showed that BAL recruited macrophages of acute CS-exposed mice, identified by their large uni-lobulated DAPI-stained nucleus, featured high levels of active processed caspase-1 in comparison to BAL cells from air-exposed mice (**Figures 5A, B**). In addition, BAL recruited neutrophils, identified by their tri-lobulated nucleus and small size compared to macrophages presented a weak expression of cleaved caspase-1. In contrast, cleaved caspase-1 was almost not detectable in BAL alveolar macrophages from MCC950-treated acute CS-exposed WT mice or from CS-exposed *Nlrp3^-/-^* mice (**Figures 5A, B**). These results indicate that acute CS-exposure promotes NLRP3-dependent caspase-1 activation in macrophages and recruited neutrophils of the bronchoalveolar space.

### CS exposure promotes NLRP3-dependent activation of gasdermin D in lung tissue

**Figure 6:**
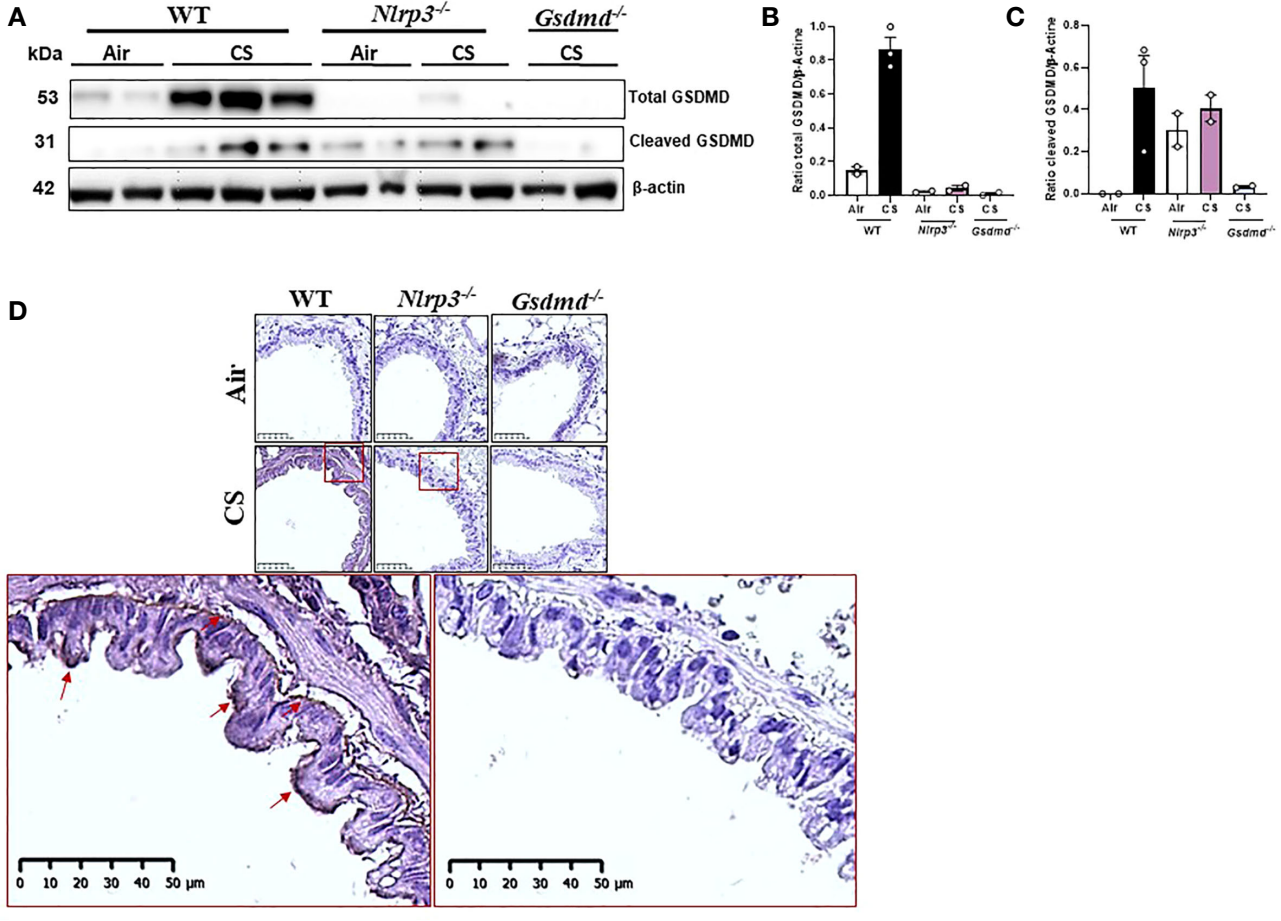
Cigarette Smoke (CS) exposure induces GSDMD cleavage in bronchial epithelial cells in an NLRP3-dependent manner. Immunoblot for total and cleaved GSDMD proteins in lung homogenates of Air or CS-exposed mice of WT, *Nlrp3^-/-^* or *Gsdmd^-/-^* mice **(A)** and quantification of total GSDMD **(B)** and cleaved GSDMD **(C)** immunoblot were shown. Cleaved GSDMD immunostaining was performed on lung sections of Air or CS-exposed WT, *Nlrp3^-/-^* and *Gsdmd^-/-^* mice **(D)**. Data are representative of three experiments. Bar graph are expressed ± SEM (n =2–4 mice per group).

To better understand the mechanisms of inflammation in response to acute CS-exposure, we investigated whether NLRP3 activation leads to gasdermin D (GSDMD) cleavage and possibly to IL-1β secretion or pyroptosis, an immunogenic cell death (ICD) associated with NLRP3 activation. GSDMD is a 53.2 kDa protein that is cleaved by caspase-1 and/or caspase-11 upon NLRP3 activation, resulting in the generation of the GSDMD-N p31 fragment. The active GSDMD-N forms pores in the plasma membrane, allowing small cytokine release such as mature IL-1β and/or ion fluxes that may lead to cell swelling and to pyroptosis. We first analysed the level of the p53 full-length GSDMD and/or the GSDMD-N p31 active form in the lungs of acute CS-exposed mice by western blot. We observed that acute CS exposure increased lung tissue expression of the pro-GSDMD p53 in WT mice that was strongly reduced in acutely CS-exposed *Nlrp3^-/-^* mice (**Figures 6A, B**; **Figure S2**). Importantly, using an antibody specifically recognizing the cleaved form of GSDMD at its N-terminal domain, we show that acute CS-exposure induced lung expression of the cleaved GSDMD p31 protein in WT mice but less importantly in lungs of *Nlrp3^-/-^* acute CS-exposed mice (**Figures 6A-C**; **Figure S2**). Furthermore, lung section immunostaining analysis indicates that acute CS induced expression of the cleaved GSDMD p31 in bronchial airway epithelial cells that was reduced in lung of *Nlrp3^-/-^* mice and absent in *Gsdmd^-/-^* mice as compared to WT mice (**Figure 6D**). However we did not observe cleaved GSDMD expression in lung alveolar regions or in infiltrating cells that were not detectable on histology slides after only 4 days of CS exposure. These results demonstrate that acute CS exposure induces NLRP3 activation which is involved in GSDMD protein cleavage in bronchial airway epithelial cells.

### Mouse CS exposure induces NLRP3-dependent activation of GSDMD in bronchoalveolar space macrophages

**Figure 7:**
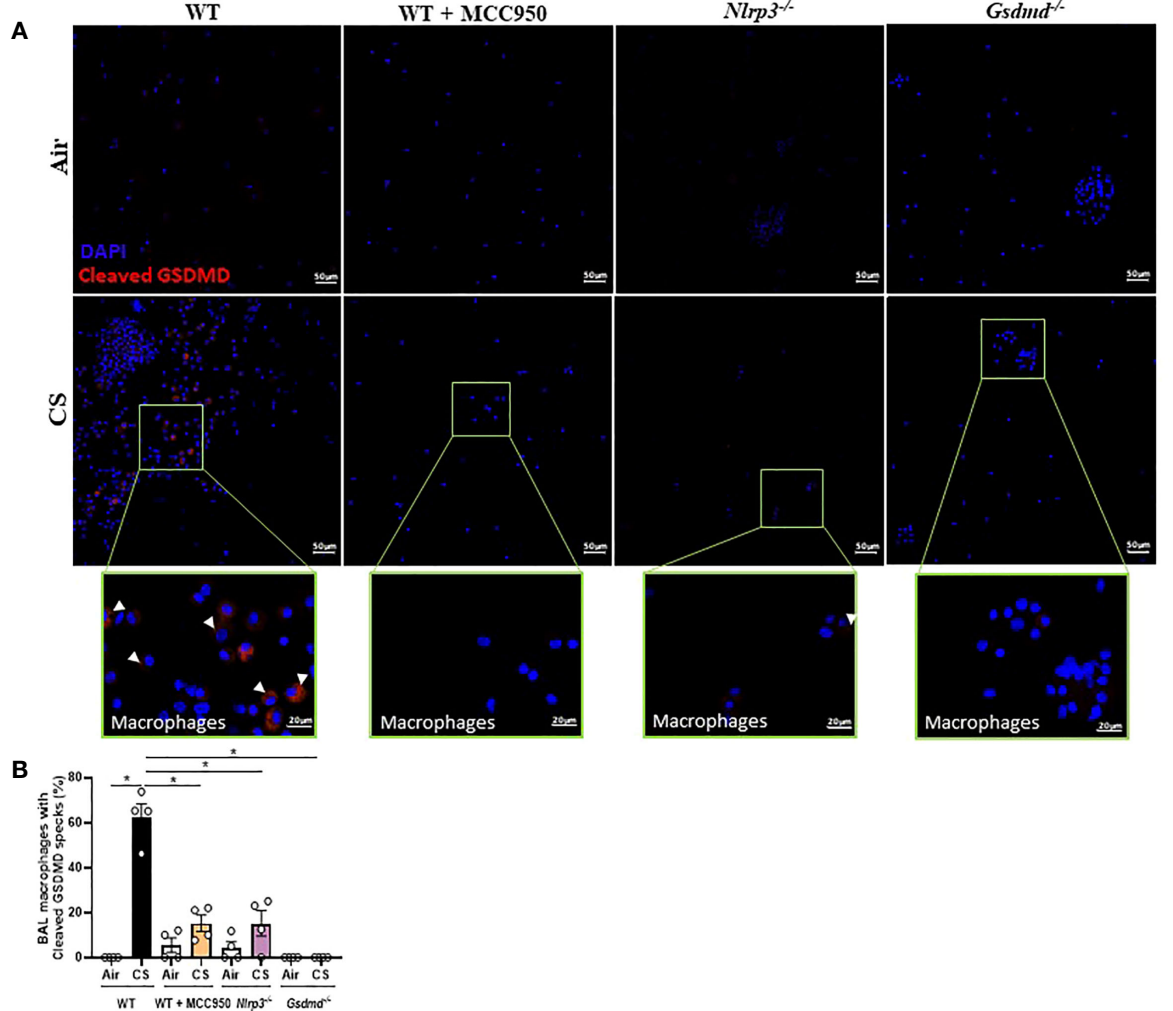
Cigarette Smoke (CS) exposure promotes GSDMD expression and cleavage in BAL alveolar macrophages dependently of NLRP3. Cleaved GSDMD immunostaining on BAL cells collected after Air or CS exposure in WT, MCC950-treated WT mice, *Nlrp3^-/-^* and *Gsdmd^-/-^* mice. Cleaved GSDMD is shown in red and nucleus in blue (DAPI) **(A)**. Airway macrophages fluorescence intensity quantification **(B)**. Data are representative of three experiments. Bar graph is expressed as mean ± SEM (n =2–4 mice per group, *p < 0.05 using a Mann Whitney test).

We next determined whether GSDMD activation occurs in BAL cells upon CS exposure by immunostaining using the specific antibody that recognizes the cleaved N-terminal domain of GSDMD. We observed that airway macrophages, identified by their large unit-lobulated DAPI-stained nucleus, show cleaved GSDMD in response to acute CS-exposure of WT mice but not in recruited neutrophils (**Figures 7A, B**). Importantly, cleaved GSDMD levels in macrophages were reduced in CS-exposed WT mice treated with MCC950 and importantly reduced in CS-exposed *Nlrp3^-/-^* mice (**Figures 7A, B**). These results demonstrate that CS-exposure induces NLRP3 dependent GSDMD cleavage in bronchoalveolar space macrophages potentially through NLRP3 inflammasome activation.

### GSDMD deficient mice have reduced pulmonary inflammation to CS exposure

**Figure 8:**
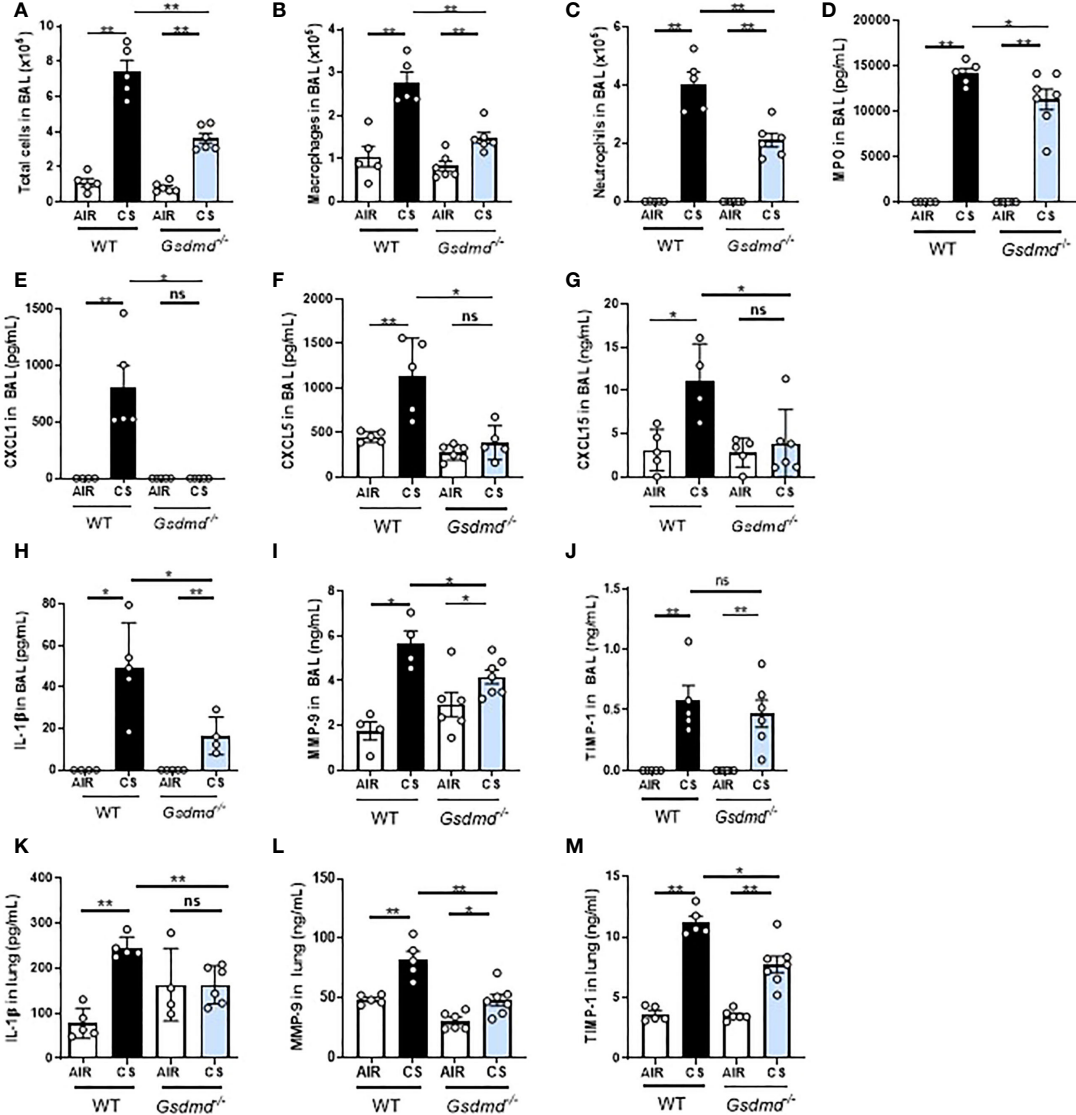
*Gasdermin d*-deficient mice (*Gsdmd^-/-^*) have reduced pulmonary inflammation and remodeling after acute smoke cigarette (CS) exposure. WT and *Gsdmd^-/-^* mice were exposed to CS or Air during 4 days. Total cells **(A)**, macrophages **(B)** and neutrophils **(C)** counts and MPO level in BAL **(D)** are shown. CXCL1 **(E)**, CXCL5 **(F)**, CXCL15 **(G)** and IL-1β **(H)** levels were measured in BAL. MMP-9 **(I)** and TIMP-1 **(J)** remodeling factor levels were measured in BAL. In lungs, levels of IL-1β **(K)**, MMP-9 **(L)** and TIMP-1 **(M)** were also measured. All parameters decreased in *Gsdmd^-/-^* mice exposed to CS as compared to CS WT mice. Data are representative of four experiments and are expressed as mean values ± SEM (n= 4-6 mice per group, *p < 0.05, **p < 0.01, ns, non significant, using a Mann Whitney test).

Finally, in order to confirm that GSDMD participates in pulmonary inflammation to acute CS exposure, we exposed WT and *Gsdmd*-deficient (*Gsdmd^-/-^*) mice to Air or CS for 4 days. We observed that total cells, macrophages and neutrophils (**Figures 8A-C**) together with MPO, CXCL1, CXCL5 and CXCL15 (**Figures 8D-G**) levels were significantly decreased in BAL of acutely CS-exposed *Gsdmd^-/-^* mice as compared to WT mice. Importantly, IL-1β levels in BAL (**Figure 8H**) was significantly reduced in *Gsdmd^-/-^* mice indicating a role of GSDMD in IL-1β release. In addition, BAL levels of MMP-9 (**Figure 8I**) and TIMP-1 (**Figure 8J**) were significantly and non-significantly reduced respectively in acutely CS-exposed *Gsdmd^-/-^* mice as compared to WT CS-exposed mice. Finally, IL-1β (**Figure 8K**) levels in lungs of acutely CS-exposed *Gsdmd^-/-^* mice was also significantly reduced, as well as MMP-9 and TIMP-1 (**Figures 8L, M**) compared to WT mice. Altogether, these results confirm that GSDMD protein is a key player of pulmonary inflammatory responses to acute CS exposure.

## Discussion

We investigated whether NLRP3 is involved in pulmonary inflammation induced by acute or subchronical experimental CS-exposure. We previously demonstrated that murine CS-induced airway inflammation depends on the IL-1R1/MyD88 signaling pathway and showed that *ex vivo* stimulation of bone marrow-derived macrophages with LPS and ATP, promotes caspase-1-dependent IL-1β maturation and secretion, suggesting involvement of an inflammasome complex (40). However, subsequent *in vivo* studies were controversial regarding the role of NLRP3 in inflammation to CS (12–15).

We demonstrate here that NLRP3 is a key player in pulmonary inflammation and remodeling upon acute or subchronical mouse CS exposure. Macrophages and neutrophils are innate immune cells infiltrating lung and recruited in bronchoalveolar space upon CS exposure and also associated with COPD pathogenesis (1, 2). Pro-inflammatory cytokines, chemokines and proteases, secreted by pulmonary recruited cells and lung resident cells amplify organ inflammation and damages. Here we highlighted a decrease of bronchoalveolar space and lung tissue cell influx, cytokines/chemokines expression and/or release upon acute and subchronic CS exposure after knockdown of the NLRP3 sensor. Influx of macrophages, and neutrophils and importantly IL-1β secretion in the bronchoalveolar space of acutely of subchronically CS-exposed mice were dependent on NLRP3. Furthermore, we report attenuated inflammation and remodeling responses to acute CS exposure after pharmacological inhibition of the NLRP3 inflammasome by MCC950 treatment. MCC950 was shown to directly targets the NLRP3 ATP hydrolysis motif to specifically inhibit canonical and non-canonical NLRP3 formation at nanomolar concentrations (38). MCC950 is active *in vivo* in multiple NLRP3-dependent mouse models, impairing IL-1β production and attenuating the severity of inflammatory disease models (37). Although we cannot exclude that MCC950 may non-specifically inhibit other targets, our data with genetic depletion and pharmacological inhibition strongly suggest that NLRP3 is a key sensor of pulmonary injury and in particular induced by acute CS exposure but possibly after other noxious airway exposure, open new fields to investigate. Importantly, MCC950 treatment might be beneficial in preventing pulmonary inflammation and this hypothesis could be tested in chronically CS-exposed mice models. However, although MCC950 inhibited the NLRP3 inflammasome in a lot of nonclinical models of inflammatory diseases with very high specificity and at nanomolar concentrations, this inhibitor presents pharmacokinetic and toxicokinetic properties that may limit its therapeutic development in the clinic. Several improved next-generation inhibitors are now in clinical trials (39). We further showed that *Caspase1/11* deficiency leads to reduced pulmonary inflammation suggesting that NLRP3-canonical and/or non-canonical inflammasome pathways are involved in acute CS exposure-induced lung inflammation. Moreover, mouse CS exposure induced cleaved caspase-1 in macrophages and neutrophils present in the bronchoalveolar space, which was dampened after knockdown or pharmacological inhibition of the NLRP3 sensor. These results demonstrated that the canonical NLRP3 inflammasome is a key player of acute CS exposure-induced inflammation mediated by macrophages and neutrophils in the bronchoalveolar space.

In addition, we investigated the role of the gasdermin D (GSDMD) in pulmonary inflammation upon mouse acute CS exposure. GSDMD-forming pores is known to drive a rapid pro-inflammatory form of cell death known as pyroptosis and release pro-inflammatory cytokines and endogenous danger signals (21–23). Importantly, GSDMD-forming pores were also identified as the most important way for the secretion of mature IL-1β (27). GSDMD activation may result in different fate such as membrane repair and cell survival depending on cell types (28, 29). Interestingly, we demonstrated that NLRP3 mediates GSDMD activation in bronchial epithelial cells and in macrophages of the bronchoalveolar space upon acute CS exposure of mice, possibly through NLRP3 inflammasome activation. Finally we demonstrated for the first time in *Gsdmd* deficient mice that this pore-forming protein is central to airway inflammation upon mouse acute CS exposure, probably through GSDMD expression and activation in bronchoalveolar space recruited macrophages and in bronchial airway epithelial cells.

Importantly, implication of GSDMD activation in acute CS-induced lung inflammation opens new potential studies in chronical CS-induced lung inflammation, using GSDMD inhibitors such as disulfiram that blocks pore formation (41). Pore forming ability of GSDMD represents a drug target to block IL-1β release during inflammasome-driven sterile inflammatory diseases.

Collectively, our data report a key role of the NLRP3 sensor and of the pore-forming GSDMD protein, in pulmonary inflammation to acute CS exposure in mice.

## Materials and methods

### Mice

Wild type C57BL/6J (WT) male mice were purchased from the Janvier Laboratory.

*Caspase1/11^-/-^* (42) were provided by Seshadri Tara at BASF Bioresearch corporation, *Nlrp3^-/-^* (10) by Dr. Jürg Tschopp and *Gsdmd^-/-^* (27) by Dr Petr Broz. Mice were backcrossed 10 times or made on C57BL/6J background. All mice were housed at the animal facility at transgenose institute (TAAM) in CNRS of Orleans, France. For experiments, adults (8-11 weeks old) were kept in sterile, isolated and ventilated cages. All animal experiments followed the French government’s ethical and animal experiment regulations (APAFIS 2020 #26177).

### Cigarette smoke model

Cigarette smoke (CS) exposure was performed using a calibrated EMKA InExpose smoking robot. Mice were exposed to mainstream cigarette smoke in a whole-body chamber for 20 min, 3 times a day for 4 days for acute model and on 5 consecutive days for 6 weeks for subchronic model. We used 3R4F research cigarettes (University of Kentucky) without filter removed and the cigarettes were puffed once per minute, 4s duration, 200 ml puff volume. The experimental bias flow, required to deliver CS and fresh air to the mice, is calibrated at 3.10^7^ L.min^-1^ and maintained constant. The concentration of smoke particulates is estimated to be 350 mg/cubic meter as mentioned by the manufacturer as previously described (32, 33). Our cigarette smoke exposure models are based on classical experimental models used to analyse the mechanism of CS-induced inflammation and/or COPD in mice (43, 44). These smoke exposure models mimic important features observed in human including epithelial injury and pulmonary inflammation with neutrophils influx (1, 2, 6).

### MCC950 mice treatment

Mice were treated intraperitoneally with 20mg/kg of MCC950 provided by Luke O’Neill (Coll, 2015 #3697), dissolved in (2-Hydroxypropyl)-β-cyclodextrin (H31133, Alfa Aesar) vehicle or with vehicle alone everyday between the first and the second CS exposition.

### Broncho-alveolar lavage (BAL)

BAL was performed as previously described (32, 33). Differential cell counts were performed by counting an average of 250 cells on cytospin preparations (Shandon CytoSpin 3, ThermoFisher Scientific™, Illkirch, France) after May-Grünwald-Giemsa staining (Diff Quick, Medion Diagnostics, Düdingen, Switzerland) according to manufacturer’s instructions.

### Lung homogenates

After BAL the lung were perfused with Isoton^®^ (Beckman Coulter France, Villepinte) to flush the vascular content. For protein analysis, the trilobed lung part was homogenized by a rotor-stator (Ultra-turrax^®^) in 1ml of T-Per protein extraction buffer (ThermoFisher Scientific™) mixed with protease and phosphatase inhibitor (ThermoFisher Scientific™). The extract was centrifuged 10 min at 10000 rpm and the supernatant was stored at -80°C before mediator measurement and immunoblotting analysis.

### Lung histology and Ly6G immunohistochemistry (IHC)

The left lobe of lung was fixed in 4% buffered formaldehyde (PFA), processed and paraffin embedded under standard conditions. Three microns sections were cut and stained with hematoxylin and eosin (HE). Cell infiltration was assessed by a semi-quantitative score on infiltrated area compared to the entire lung tissue area with increasing severity 0 (without any inflammation) to 5 (severe inflammation) by two independent observers on the HE-stained sections. Two additional slides per animal were cut and labelled with an anti-Ly6G antibody (ab238132, Abcam). Lung sections were scanned at X20 (0.452 µm/pixel) using a NanoZoomer-SQ and their whole slide image (WSI) captured using the NDP.view 2 software (both from Hamamatsu Corporation, Japan).

### Digital quantification of Ly6G IHC

Quantification of Ly6G was performed on the digitized sections using MorphoQuant™ software (Biocellvia, France), initially developed to measure lung lesions induced by pulmonary fibrosis or emphysema (45, 46). Briefly, neutrophils were quantified through positive labelling with Ly6G, using the classic diaminobenzidine. Positive elements appear in brown in the section and were quantified on the digitized sections using MorphoQuant™, a digital pathology software relying on morphometric analysis. In the Ly6G-labelled sections, all brown pixels were considered as Ly6G positive elements. The total number of positive Ly6G pixels was thus divided by the total number of pixels describing the whole sections, and the ratio was expressed in percent.

### Cleaved gasdermin D immunostaining

The left lobe of lung was fixed in 4% PFA for 72h, embedded in paraffin and sectioned at 3 µm. Lung sections were dewaxed and rehydrated, then heated 20 min at 80°C in citrate buffer 10 mM pH=6 for antigen retrieval (unmasking step). Lung sections were permeabilized in PBS 0.5% triton X-100, blocked with 5% FCS for 1h at RT and then incubated overnight with primary mouse anti-cleaved Gasdermin D (1:100, #10137, Cell Signaling). After washing, sections were incubated with the appropriate second antibody conjugated to horseradish peroxidase (1:200 anti-rabbit IgG, Sigma Aldrich^®^) in 1% FCS 1h at RT. Following washing, lung sections were incubated with HRP Substrate, DAB (Vector Laboratories^®^), following the manufacturer’s protocol. After distilled water washing, Gill hematoxyline counterstaining on lung sections was done. Then, lung sections were dehydrated, fixed and mounted onto microscope slides (Eukitt). Slides were examined by using a scanner NDP view.

### Mediator measurements

For cytokine determination, BAL supernatant and lung homogenates were analysed by ELISA assay kits for murine: CXCL1, CXCL5, CXCL15, MPO, LCN-2, MMP-9, TIMP-1 (Mouse DuoSet, R&D system, Minneapolis, USA) according to manufacturer’s instructions. IL-1β mesurment in BAL was measured using multiplex immunoassay according to manufacturers’ instructions (ProcartaPlex, Invitrogen™). Data were acquired on Luminex equipment (MagPix, BioRad) and analyzed using Bioplex Manager software (BioRad).

### Quantitative RT-PCR

RNA was purified from lung homogenates by using Trizol extraction protocol. Reverse transcription of RNA into cDNA was carried out with Go script reverse kit (Promega). RT-PCR was performed with Fast SYBR Green master mix on an ARIA MX (Stratagene MX3005P, Agilent technologies). Primers were synthetized (Qiagen, Hilden, Germany). The expression of *pro-IL-18* and *Mmp12* mRNA, relative to housekeeping 18S mRNA, were analyzed using Quantitect gene expression assays (Qiagen). For all experiments, biological quadruplicate and technical triplicate were performed.

### Immunoblotting

Protein concentrations were determined in lung tissue by using DC™ protein Assay (BIO-RAD, France). 40 µg of proteins were denatured by boiling (95°C, 5 min) in reducing SDS sample buffer. Samples were resolved on 13% polyacrylamide gel and run at 160V for 45min using the Mini gel Tank (ThermoFisher Scientific™). Total protein were immunoblotted to 0.2 µm nitrocellulose membrane (Amersham™) using a Trans-Blot SD Transfer System (Bio-Rad) at 100V for 45 min. Successful protein transfer was confirmed by using Ponceau S staining. Membranes were blocked 2 hours with 5% non-fat milk (Cell signaling) in 1X TBS- 0,1% Tween^®^ 20 (20 mM Tris Trizma^®^ Base, 150 mM sodium chloride, and 0.05% Tween^®^ 20 pH 7.6) at room temperature. Then, membranes were incubated with primary antibodies, mouse anti-Gasdermin (1:500, ab209845, Abcam), anti-cleaved Gasdermin (1:500, #10137, Cell Signaling) in 5% non fat dried milk in 1X TBS- 0,1% Tween^®^ 20, over nigh at 4°C. Mouse anti-actin (1:10 000, #A3854; Sigma-Aldrich) was incubated 1h at room temperature. Membranes were then washed 3 times in 1X TBS- 0,1% Tween^®^ 20 and incubated with the appropriate second antibody conjugated to horseradish peroxidase (anti-rabbit IgG, Sigma-Aldrich) 1h at room temperature. Protein bands were visualized following exposure of the membrane to Amersham ECL™ prime substrate solution using PXi gel doc system^®^ Syngene, and quantified using ImageJ software.

### Immunostaining on Cytospin

Cytospin slides were fixed in 4% PFA (Sigma-Aldrich). Cells were washed 3 times in TBS, incubated 15 min in TBS-0.3% Triton X-100, then washed 3 times in TBS, blocked in TBS-10% Bovine Serum Albumin (BSA) for 45min and incubated overnight with primary rabbit anti-Cleaved Caspase1 (1:100, #89332, Cell Signaling) or anti-Cleaved Gasdermin (1:100, #10137, Cell Signaling). After washing, cytospins were incubated with goat anti-rabbit IgG secondary antibodies conjugated with Alexa^®^ Fluor 594 (1:200, # A-11037, Invitrogen) in 1% FCS. Following washing, cells were stained with DAPI (1:2000) for 5 min, washed with PBS, and mounted onto microscope slides (Fluoromount). Immunofluorescence staining were blindly examined by using a Leica microscope at 20X magnification (Leica, Solms, Germany). Images were treated using Zen blue Zeiss software. Quantifications were realized at least for 2 mice per group, on different cytospin spots for each one.

### Statistical analysis

Statistical tests of selected populations were performed using Mann-Whitney non-parametric test. Data represent mean ± SEM. P values <0.05 were considered statistically significant.

## Data availability statement

The raw data supporting the conclusions of this article will be made available by the authors, without undue reservation.

## Ethics statement

The animal study was reviewed and approved by APAFIS N°26177-2019021818223038v15.

## Author contributions

SH-M, MN, EC, MB, AG, FS, CP, and NR performed the experiments. SH-M, MN, AG, and IC, conceived the experiments and analyzed the data. ML supervised the breeding of knock-out mice. PB provided *Gsdmd* deficient mice. SH-M, AG, VQ, BR, PB, NR, CS, and IC discussed the results. SH-M, AG, NR, PB, and IC wrote the manuscript and IC and VQ provided funding and IC overall supervision of this study.

## Funding

Grant support by the «Agence nationale de Recherche» (ANR AAPG2019 CES15 Smoke6) and the “Fondation pour la Recherche Médicale” (EQU202003010405) and the Centre National de Recherche Scientifique (CNRS) and the University of Orleans. This work was supported by CNRS, University of Orleans, the «Agence nationale de Recherche» (ANR AAPG2019 CES15 Smoke6) and the “Fondation pour la Recherche Médicale” (EQU202003010405).

## Acknowledgments

We also thank Luke O’Neill for providing us with the MCC950 inhibitor. We also thank Nathalie Froux, Pauline Abgrall, and all technicians from our specific pathogen-free animal facility at CNRS (TAAM UPS44, Orléans, France) for breeding and animal care.

## Conflict of interest

The authors declare that the research was conducted in the absence of any commercial or financial relationships that could be construed as a potential conflict of interest.

## Publisher’s note

All claims expressed in this article are solely those of the authors and do not necessarily represent those of their affiliated organizations, or those of the publisher, the editors and the reviewers. Any product that may be evaluated in this article, or claim that may be made by its manufacturer, is not guaranteed or endorsed by the publisher.

## Supplementary material

Supplementary Figure 1
*Nlrp3*-deficient mice (*Nlrp3^-/-^*) have reduced pulmonary inflammation after acute cigarette smoke (CS) exposure. WT and *Nlrp3^-/-^* mice were exposed to CS or Air during 4 days **(A)**. Total cells **(B)**, macrophages **(C)**, neutrophils **(D)** counts and MPO **(E)**, CXCL1 **(F)**, IL-1β **(G)** and remodeling factors MMP-9 **(H)** and TIMP-1 **(I)** levels in BAL were decreased in *Nlrp3^-/-^* mice exposed to CS compared to CS WT mice. CXCL1 **(J)**, IL-1β **(K)** and MMP-9 **(L)** and TIMP-1 **(M)** levels in lungs are shown. Data are representative of four experiments and are expressed as mean values ± SEM (n= 4-6 mice per group, *p < 0.05, **p < 0.01, using a Mann Whitney test).

Supplementary Figure 2 Total red ponceau membrane. Total protein migration was verified on total membrane using red ponceau staining for the following immunoblot analysis. Data are representative of three experiments.
