# Supplementary figures and images for: Cigarette smoke-induced gasdermin D activation in bronchoalveolar macrophages and bronchial epithelial cells dependently on NLRP3
Source: Front Immunol. 2022 Aug 15;13:918507. doi: 10.3389/fimmu.2022.918507 (PMC9421433; doi:10.3389/fimmu.2022.918507)

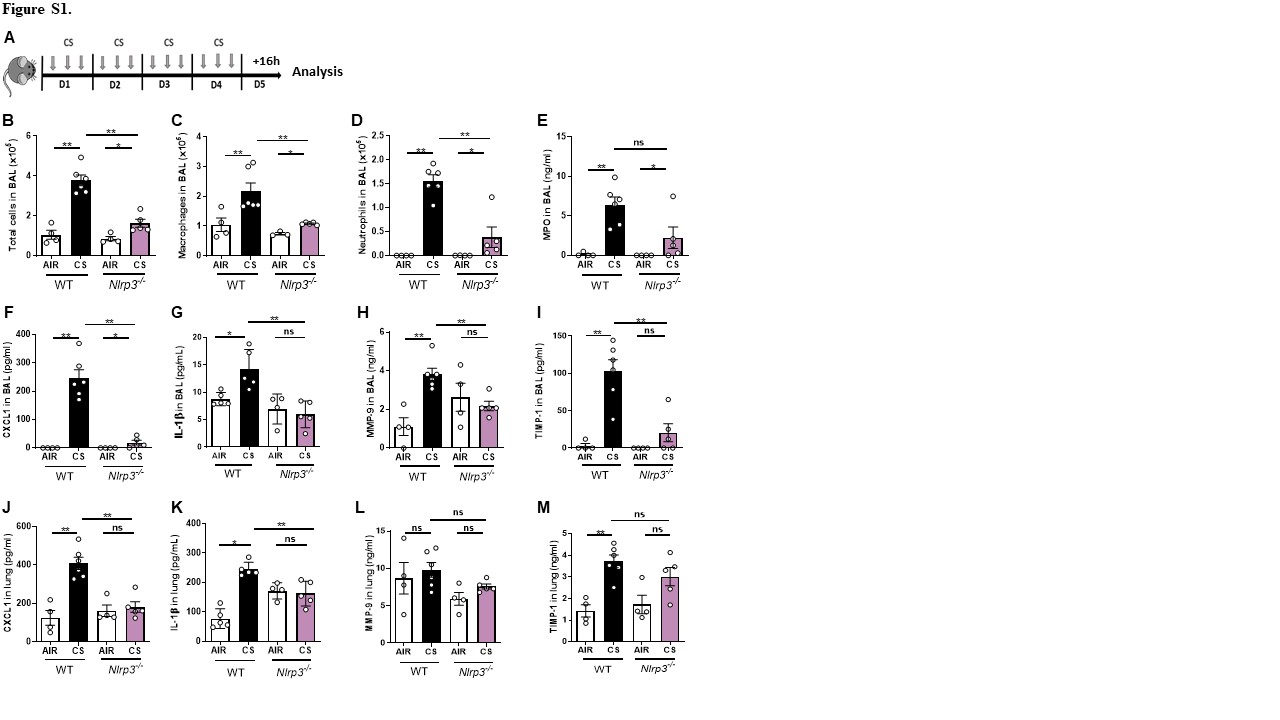

Supplement: Supplementary Figure 1 — Nlrp3-deficient mice (Nlrp3-/- ) have reduced pulmonary inflammation after acute cigarette smoke (CS) exposure. WT and Nlrp3-/- mice were exposed to CS or Air during 4 days (A). Total cells (B), macrophages (C), neutrophils (D) counts and MPO (E), CXCL1 (F), IL-1β (G) and remodeling factors MMP-9 (H) and TIMP-1 (I) levels in BAL were decreased in Nlrp3-/- mice exposed to CS compared to CS WT mice. CXCL1 (J), IL-1β (K) and MMP-9 (L) and TIMP-1 (M) levels in lungs are shown. Data are representative of four experiments and are expressed as mean values ± SEM (n= 4-6 mice per group, *p < 0.05, **p < 0.01, using a Mann Whitney test). [file Image_1.jpeg]

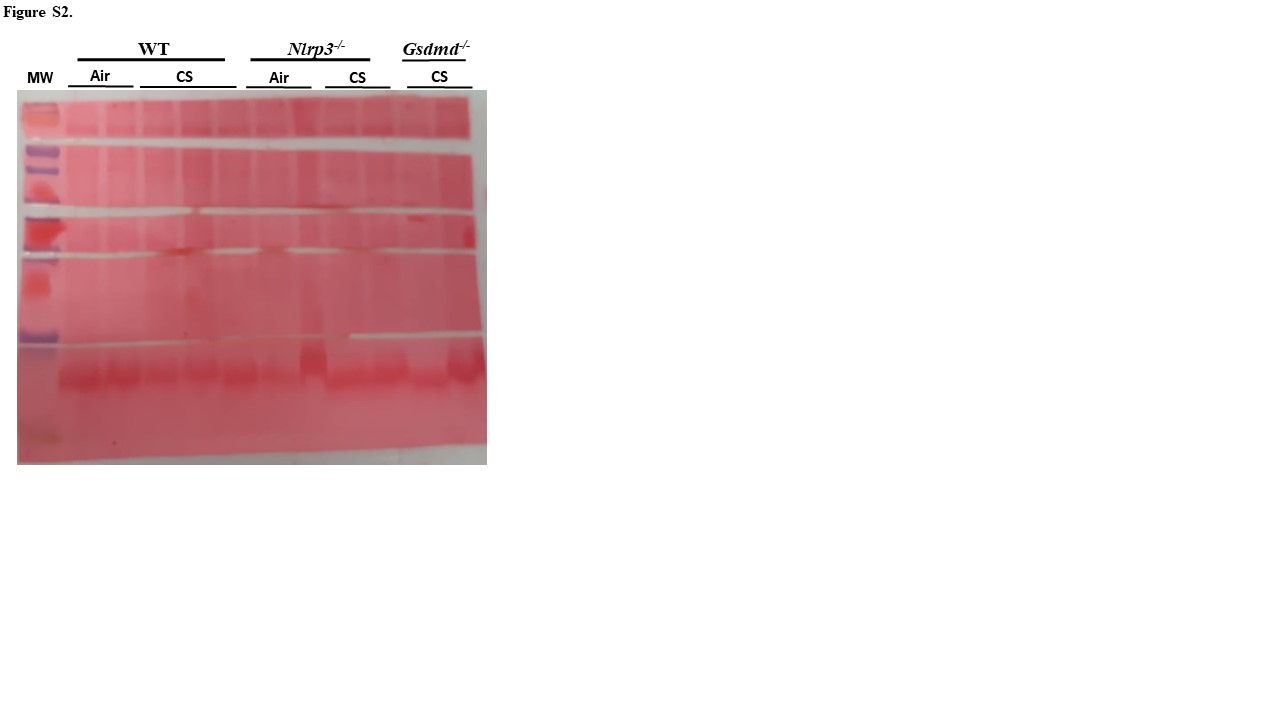

Supplement: Supplementary Figure 2 — Total red ponceau membrane. Total protein migration was verified on total membrane using red ponceau staining for the following immunoblot analysis. Data are representative of three experiments. [file Image_2.jpeg]
